# Fat-suppressed T2 mapping of human knee femoral articular cartilage: comparison with conventional T2 mapping

**DOI:** 10.1186/s12891-021-04542-9

**Published:** 2021-08-09

**Authors:** Bo Ram Kim, Hye Jin Yoo, Hee-Dong Chae, Sung Hwan Hong, Ja-Young Choi

**Affiliations:** 1 Department of Radiology, Seoul National University Bundang Hospital, Seongnam-si, Korea; 2 Department of Radiology, Seoul National University Hospital, 101 Daehak-ro, Jongno-gu, 03080 Seoul, Korea

**Keywords:** MRI, Cartilage, Knee, T2 mapping, Fat suppression technique, Relaxomatry

## Abstract

**Background:**

There is paucity of studies applying fat suppressed (FS) technique to T2 mapping to overcome chemical shift artifacts. The purpose of the study is to difference between FS T2 and conventional T2 mapping and reproducibility of FS T2 mapping in the femoral articular cartilage.

**Methods:**

Eighteen patients who had normal-looking femoral cartilage and underwent knee MRI with conventional T2 and FS T2 mapping were included. T2 values of each mapping were measured by two readers independently from nine regions in the medial femoral condyle (MFC) and lateral femoral condyle (LFC). Each anatomical region was divided by lines at ± 10°, 30°, 50°, 70°, 90°, and 110°. Comparisons of T2 values between conventional and FS T2 mapping were statistically analyzed. The T2 values between FS and conventional T2 mapping in the anterior, central and posterior femoral condyles were compared.

**Results:**

The overall femoral condyle T2 values from the FS T2 map were significantly lower than those from the conventional T2 map (48.5ms vs. 51.0ms, *p* < 0.001). The differences in the T2 values between the two maps were significantly different among the three divisions of the LFC (*p* = 0.009) and MFC (*p* = 0.031). The intra-class correlation coefficients indicated higher agreement in the FS T2 map than in the conventional T2 map (0.943 vs. 0.872).

**Conclusions:**

The T2 values of knee femoral cartilage are significantly lower on FS T2 mapping than on conventional T2 mapping. FS T2 mapping is a more reproducible method for evaluating knee femoral cartilage.

## Background

Magnetic resonance (MR) imaging is a widely used imaging modality for assessing morphological changes in articular cartilage. However, this modality has limitations in assessing compositional and ultrastructural changes [1, 2], which are needed for the detection of early-stage osteoarthritis. In this regard, several more advanced compositional MR imaging techniques (T2 mapping, T2* mapping, T1rho) have been developed and demonstrated to identify composition changes preceding morphological changes in the cartilage [3–5]. Among them, T2 mapping, which calculates the T2 relaxation values of the cartilage from mulit-echo spin-echo based sequences, is a well-documented technique regarding the reproducibility and validity of T2 quantification and is sensitive to the degradation of the collagen matrix [6–8]. An increased T2 relaxation time on T2 mapping represents cartilage degeneration and is used as a quantitative parameter for monitoring biochemical changes in the cartilage in response to treatment. For this reason, T2 mapping gathered much attention as a quantitative imaging biomarker and, moreover, has been introduced in clinical practice as a routine MR imaging sequence [9–12]. Therefore, the optimization of and reduction in potential diagnostic error sources is emphasized for improving the accuracy of cartilage T2 mapping.

Chemical shift artifacts are a potential source of error on T2 mapping, occurring at the bone-cartilage interface and cartilage-intraarticular fat tissue interface. This can affect the measurement of the T2 relaxation time and the thickness of articular cartilage, especially on 3T MR imaging and for knee joints [13, 14]. The effect of chemical shift artifacts can be minimized by changing the frequency encoding direction. However, this produces not only pulsating artifacts but also chemical shift artifacts in other regions of the knee cartilage. The fat suppression technique is an alternative solution for eliminating chemical shift artifacts and improving cartilage delineation. To date, there have been a paucity of studies applying the fat suppression technique to T2 mapping as a method to overcome the chemical shift artifacts of conventional T2 mapping of knee cartilage. According to previous ex vivo and in vivo studies with a small sample of healthy volunteers [15, 16], fat-suppressed (FS) T2 mapping helps improve reproducibility by eliminating the chemical shift artifact. Therefore, the purpose of our study is to analyze the difference between FS T2 mapping and conventional T2 mapping and the reproducibility of FS T2 mapping in the knee femoral articular cartilage.

## Methods

### Study population

The review board of Seoul national university hospital approved this retrospective study and the requirement for patient informed consent was waived. All methods were carried out in accordance with guidelines and regulations of committee on ethics. From April 2015 to August 2016, we included patients who had normal-looking femoral articular cartilage and underwent a knee MR imaging protocol with a conventional T2 mapping sequence and an FS T2 mapping sequence at the same time. In total, eighteen patients (M:F = 10:8, mean age = 46.2 ± 14.8 years) were included in the study. Among the 18 patients, 4 (22.2 %) had normal knee structures, 5 (27.8 %) had a medial meniscus (MM) tear, 2 (11.1 %) had an anterior cruciate ligament (ACL) tear, 2 (11.1 %) had a combination of MM and ACL tears, 1 (5.6 %) had discoid meniscus and 4 (22.2 %) had cartilage defects other than at the femoral condyles (patella and trochlear groove).

### MR imaging acquisition

MRI examinations were performed using a 3T MR system (Magnetom Skyra; Siemens Healthineers, Erlangen, Germany) with a dedicated knee coil. The protocol consisted of routine clinical sequences followed by two sagittal T2 mapping sequences. Routine knee MR imaging consisted of turbo spin-echo proton density weighted images [axial, FS sagittal, and coronal planes], turbo spin-echo T2-weighted imaging (sagittal, FS coronal, and oblique coronal planes) and turbo spin-echo T1-weighted sagittal imaging.

After completion of the routine knee MR imaging, two sagittal conventional and FS T2 mapping sequences were performed using following protocols. T2 maps were generated using inline calculation software (MapIt; Siemens Healthineers) with a mono-exponential fitting from multi-slice, multi-echo source images with five different echo times (13.8, 27.6, 41.4, 55.2 and 69.0 msec). Frequency encoding was in the feet-to-head direction. The parameters used for the T2 mapping sequences are as follows: repetition time, 2000 msec for conventional T2 maps and 2300 msec for FS T2 maps; field of view, 159 mm $\times$ 159 mm; matrix 384 $\times$ 384; slice thickness, 3 mm; flip angle, 180$^\circ$; number of signals averaged, 1; bandwidth, 150 Hz/pixel; echo train length, 5; slices, 25. For FS T2 mapping, the chemical shift-selective suppression (CHESS) method was used. The generated T2 maps automatically transferred from MR scanner to picture archiving and communication systems (PACS) (Gx; Infinitt Healthcare, Seoul, Republic of Korea)

### T2 relaxation time measurement

**Fig. 1:**
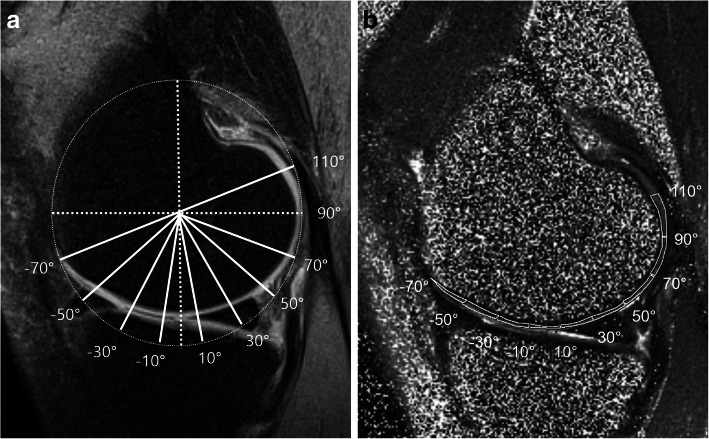
Nine ROIs drawn on femoral cartilage T2 mapping. Dashed lines indicates diameter line of circle connected two most protruding points and it’s bisecting perpendicular lines. Lines drawn at the angle of ± 10°, 30°, 50°, 70°, + 90° and + 110°, respective to perpendicular line. ROIs were drawn on anatomical T2 weighted source image (**a**) including full thickness cartilage defined by reference lines and matched to the T2 mapping (**b**)

A research assistant under the supervision of a musculoskeletal radiologist (with 17 years of experience) and a musculoskeletal radiologist (with 5 years of experience) measured the T2 relaxation time on conventional and FS T2 mapping. The T2 relaxation times were measured at nine anatomical regions (-70° ~ -50°, -50° ~ -30°, -30° ~ -10°, -10° ~ 10°, 10° ~ 30°, 30° ~ 50°, 50° ~ 70°, 70° ~ 90° and 90° ~ 110°) in the mid-sagittal image of the medial femoral condyle (MFC) and in the mid-sagittal image of the lateral femoral condyle (LFC). Each anatomical region in the MFC and LFC was divided by lines at ± 10, 30, 50, 70, 90, and 110 degrees relative to a vertical line bisecting the femoral condyle width and connecting the two most protruding points of the femoral condyle. Regions of interest (ROIs) for the T2 values were drawn in all regions along the bone-cartilage interface and cartilage surface to include the full thickness of the cartilage. The T2-weighted sagittal source image that most clearly depicted cartilage with high tissue contrast was selected among the multi-echo time source images for ROI segmentation over the cartilage. ROIs were independently and manually drawn on conventional and FS-T2 weighted source image using segmentation tool in PACS workstation. After then, ROIs were transferred to the conventional and FS T2 maps in a copy-and-paste manner, respectively (Fig. 1). Mean T2 value of the ROIs on the T2 mapping were calculated on PACS.

### Statistical analyses

The mean T2 values obtained from the two readers were used for statistical analyses. To compare the mean T2 values and ROI areas of the conventional and FS T2 maps for each region, paired t-tests were used. The relationship between the T2 values from the conventional and FS T2 maps was assessed by Pearson’s correlation analysis. One-way ANOVA was performed to determine statistically significant differences in subtracted T2 values, obtained by subtracting the FS T2 map from the conventional T2 map in each of three divisions (anterior, central and posterior). The central division was defined as the cartilage in contact with the meniscus and corresponded to -30° to 50° in the LFC and − 50° to 50° in the MFC. The anterior division was defined as the cartilage anterior to the central division, and the posterior division was defined as the cartilage posterior to the central division. Interreader and intrareader agreement for the T2 values measured on conventional and FS T2 mapping was assessed with the intraclass correlation coefficient (ICC). Additionally interreader agreement for ROI cross-sectional area also assessed with ICC. The degree of agreement was interpreted as excellent if the ICC was above 0.75, as fair-to-good (moderate) if the ICC was between 0.40 and 0.75, and as poor if the ICC was less than 0.40. The SPSS 25.0 software package (Chicago, IL, USA) was used for statistical analyses in our study, and a p-value of < 0.05 was considered to indicate statistical significance.

## Results

**Fig. 2:**
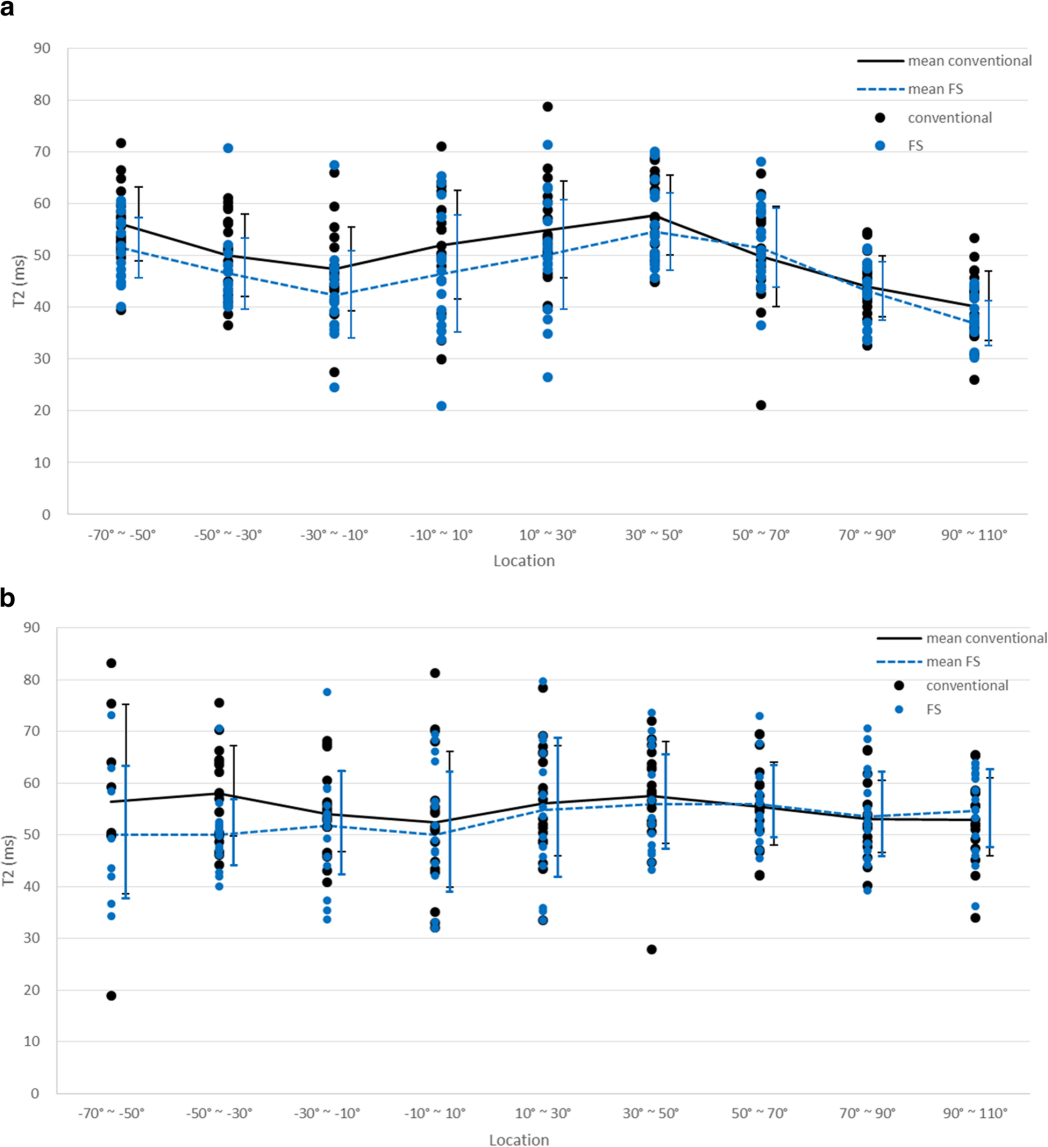
Line graphs mean value overlaid on scatter plots of conventional and FS T2 mapping, obtained from LFC (**a**) and MFC (**b**). The graphs showed lower T2 values of FS T2 mapping in the anterior and central division of LFC and MFC

**Fig. 3:**
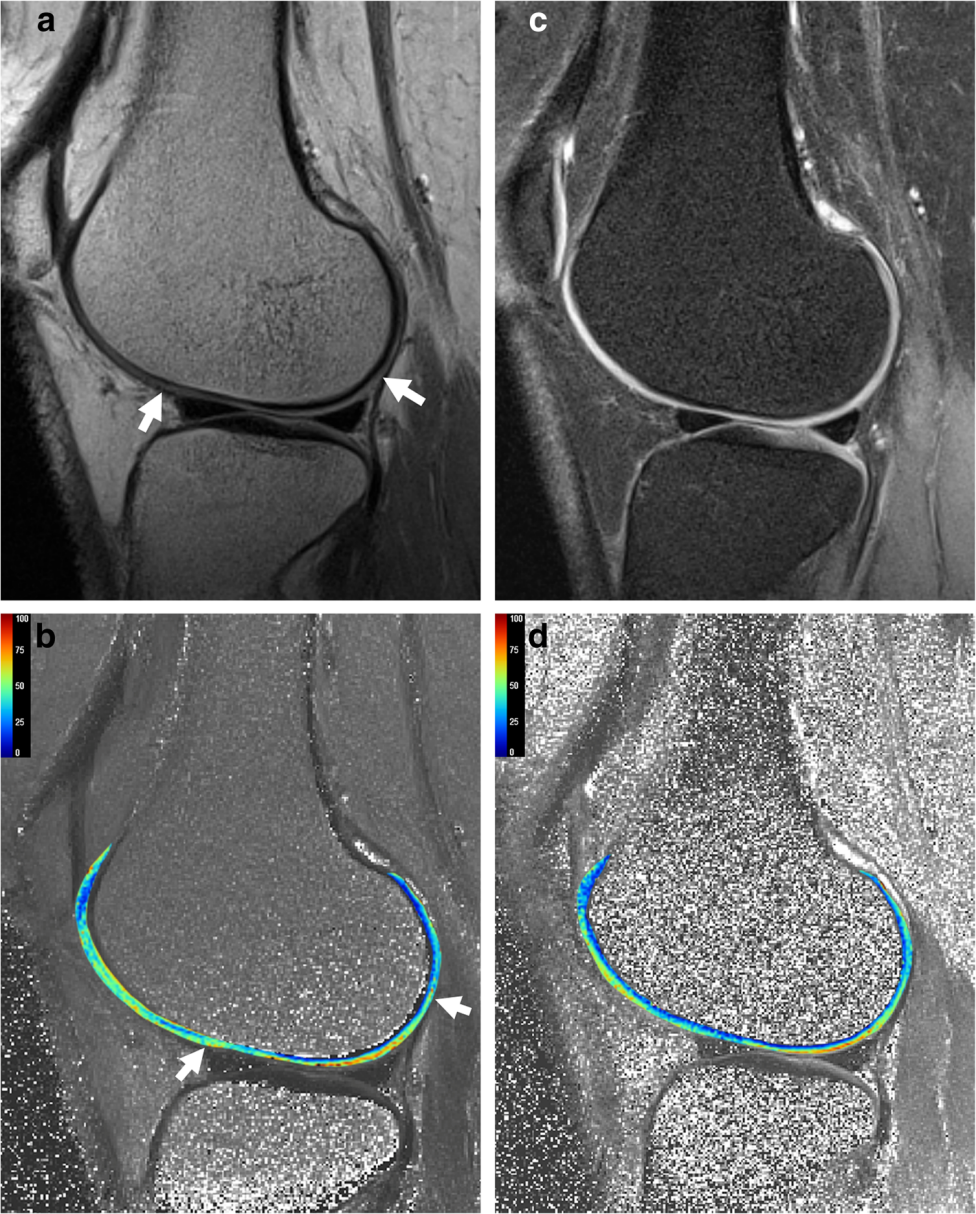
Representative conventional and FS T2 weighted LFC anatomical source images and T2 mapping of 37-years-old women. Average LFC T2 value of FS T2 mapping was lower than conventional T2 mapping (51.0 vs. 47.7). Conventional source image (**a**) and T2 mapping (**b**) showed chemical shift artifacts (arrows) on the cartilage-bone interface which affected T2 value estimation. FS source image (**c**) and FS T2 mapping (**d**) demonstrated devoid of chemical shift artifacts and clearly visualized cartilage –bone interface

The mean T2 relaxation values of the conventional and FS T2 map in the MFC and LFC are summarized in Table 1 and Fig. 2. The mean overall T2 values of the FS T2 map (48.5 ± 13.4 ms) were significantly lower than those of the conventional T2 map (51.0 ± 13.6 ms) (*p* < 0.001). In the LFC and MFC, the mean T2 values of the FS T2 map were also lower than those of the conventional T2 map (47.0 ± 9.9 ms vs. 50.3 ± 9.9 ms, *p* < 0.001 for the LFC; 49.9 ± 16.1 ms vs. 51.7 ± 16.6 ms, *p* < 0.001 for the MFC) (Fig. 3). Pearson’s correlation coefficients showed a significant positive correlation between conventional T2 and FS T2 mapping (*r* = 0.809, *p* < 0.001).

**Table 1:** Comparison of T2 values between conventional T2 mapping and fat-suppressed T2 mapping

	Conventional T2 (Mean ± SD)	Fat-suppressed T2 (Mean ± SD)	*P*-value
***LFC***			
* -70° ~ -50°*	56.1 ± 7.3	51.5 ± 6.0	**0.020**
* -50° ~ -30°*	50.0 ± 8.2	46.6 ± 7.1	0.051
* -30° ~ -10°*	47.4 ± 8.3	42.4 ± 8.7	**0.001**
* -10° ~ 10°*	52.1 ± 10.7	46.4 ± 11.6	**0.040**
* 10° ~ 30°*	55.0 ± 9.6	50.1 ± 10.9	0.077
* 30° ~ 50°*	57.8 ± 7.9	54.6 ± 7.7	**0.044**
* 50° ~ 70°*	49.8 ± 9.9	51.5 ± 7.9	0.458
* 70° ~ 90°*	44.0 ± 6.1	43.1 ± 5.8	0.541
* 90° ~ 110°*	40.3 ± 6.6	38.9 ± 10.2	0.091
***MFC***			
* -70° ~ -50°*	56.4 ± 19.5	50.1 ± 13.7	0.090
* -50° ~ -30°*	58.0 ± 9.0	50.0 ± 6.6	**0.001**
* -30° ~ -10°*	54.0 ± 8.0	51.8 ± 10.3	0.352
* -10° ~ 10°*	52.5 ± 13.5	50.1 ± 12.0	0.453
* 10° ~ 30°*	56.2 ± 11.0	54.9 ± 13.8	0.490
* 30° ~ 50°*	57.7 ± 10.1	56.0 ± 9.4	0.499
* 50° ~ 70°*	55.5 ± 8.3	56.0 ± 7.1	0.716
* 70° ~ 90°*	53.1 ± 7.2	53.5 ± 8.4	0.681
* 90° ~ 110°*	53.0 ± 7.8	54.7 ± 7.7	0.040

Note. – *SD *standard deviation, *LFC *lateral femoral condyle, *MFC *medial femoral condyle

In the LFC, the mean T2 relaxation values of the FS T2 map were lower than those of the conventional T2 map in most regions, except for the 50° ~ 70° region. The differences between conventional T2 mapping and FS T2 mapping were significant in the − 70° ~ 50° (*p* = 0.020), -30° ~ -10° (*p* = 0.001), -10° ~ 10° (*p* = 0.040), and 30° ~ 50° (*p* = 0.044) regions. Most of the differences were shown in the central division of the LFC. Similarly, the mean FS T2 values were lower than the conventional T2 values in the anterior and central divisions of the MFC. However, the posterior division of the MFC showed higher FS T2 values than conventional F2 values. Significant differences between the two T2 maps were found in the − 50° ~ -30° (*p* = 0.001) and 90° ~ 110° (*p* = 0.04) regions.

A comparison of the differences in the T2 values between the conventional T2 map and FS T2 map across the three divisions of the MFC and LFC showed significant differences (*p* = 0.031 and *p* = 0.009, respectively) (Table 2). In the LFC, the largest difference was shown in the central division, followed by anterior and posterior division (4.7 ms, 4.0 ms and 0.9 ms, respectively). In the MFC, the anterior division showed the largest difference, followed by central division and posterior division (6.3 ms, 3.1 ms and − 0.9 ms, respectively).

**Table 2:** Comparison of T2 values difference between conventional and fat-suppressed T2 map in three divisions

	Anterior	Middle	Posterior	*P*-value*
LFC	4.0 ± 7.2	4.7 ± 8.5	0.9 ± 8.1	0.031†
MFC	6.4 ± 9.1	3.1 ± 10.2	− 0.9 ± 4.3	0.009†

Note. – *LFC *lateral femoral condyle, *MFC *medial femoral condyle

*P values were determined by Tukey post hoc comparisons

†Significant difference between middle and posterior divisions

The inter-reader agreement for the T2 values of both maps indicated almost perfect agreement. However, the ICC indicated higher agreement in the FS T2 map than in the conventional T2 map (0.943 vs. 0.872) for the overall cartilage T2 value. The intra-reader agreement for the T2 values of both demonstrated perfect agreement. The ICC indicated also indicated higher agreement in the FS T2 map than in the conventional T2 map (0.945 vs. 0.986).

Additionally, the ROI cross-sectional areas for each region showed higher interreader agreement in the FS T2 map than in the conventional T2 map (0.910 vs. 0.894). The mean cross-sectional area of the ROI was larger on FS T2 mapping than on conventional T2 mapping (8.08 ± 4.90 mm^2^ vs. 7.26 ± 4.53 mm^2^, *p* = < 0.001).

## Discussion

In this study, the mean T2 values of the femoral articular cartilage on FS T2 mapping were lower than those of conventional T2 mapping. In particular, the anterior and central divisions of the MFC and LFC showed consistently lower T2 values on FS T2 mapping than on conventional T2 mapping. Moreover, FS T2 mapping of the femoral articular cartilage yielded better interobserver agreement for the T2 values and ROI areas than conventional T2 mapping.

Previous studies [15, 16] suggested that compared with that on conventional T2 mapping, the T2 value is reduced on in F2 T2 mapping of the knee articular cartilage. Rye et al. [16] demonstrated significantly lower T2 values on FS T2 mapping than on conventional T2 mapping with porcine knees. Paakkari et al. [15], similar to previous ex vivo results, demonstrated lower T2 values on FS T2 mapping than on conventional T2 mapping in healthy human volunteers. As in our results, the mean T2 values of FS T2 mapping shifted downward from those of conventional T2 mapping in the anterior and central divisions despite regional differences. Downward-shifted T2 values on FS T2 mapping might reflect a reduction in fat contamination and chemical shift artifacts by the fat suppression technique. The signal arising from the fat tissue around the knee, that is, the infrapatellar fat pad, which affects the anterior third of the femoral condyle cartilage, was suppressed on FS T2 mapping. Furthermore, the signal from the subchondral fatty bone marrow was suppressed, and chemical shift artifacts were eliminated from the cartilage-bone interface, which was particularly affected at the central third of the femoral condyle cartilage in the feet-to-head frequency encoding direction.

Colotti et al. [17] applied a lipid-insensitive T2 mapping technique (LIBRE-Iso3DGRE) on isotropic 3D gradient recall echo (Iso3DGRE) T2 mapping. The LIBRE-Iso3DGRE T2 values were slightly higher than the Iso3DGRE values on in vivo T2 mapping (36.5 ms vs. 34.1 ms, *p* = 0.1). The discrepancy with our results might result from the different fat suppression techniques implemented. LIBRE-Iso3DGRE applies water excitation with a novel lipid-insensitive binomial off-resonant radiofrequency excitation pulse, and our study applied the CHESS technique, which is commonly used in musculoskeletal MR imaging [18]. In addition, GRE-based pulse sequences might affect this discrepancy.

We demonstrated better reproducibility with FS T2 mapping than with conventional T2 mapping in terms of the T2 values and ROI areas. Although their changes in the T2 value on FS T2 mapping do not match our results, Colotti et al. [17] also suggested that lipid-insensitive T2 mapping allows for more accurate T2 value evaluation by eliminating chemical shift artifacts. FS T2 mapping and conventional T2 mapping showed similar cartilage imaging features. However, FS T2 mapping can remove signal overlapping and voiding phenomena from the cartilage-bone interface in some areas of the image caused by chemical shift artifacts on conventional T2 mapping, thus providing better discrimination of the cartilage margin and subchondral bone plate. In the present study, the ROI cross-sectional areas for each region were larger and showed higher interreader agreement in the FS T2 map than in the conventional T2 map. Hence, the lack of chemical shift artifacts and better cartilage contrast improves reproducibility. Therefore, we suggest that FS T2 mapping is a more reliable method than conventional T2 mapping for evaluating knee cartilage. However, to establish optimal T2 mapping methods for cartilage evaluation, further prospective studies with larger populations are warranted.

Several limitations of this study need to be mentioned. First, the study sample was small and excluded patients with chondral abnormalities on MRI. Further investigations with larger study populations, including patients with chondral abnormalities, need to be conducted. Second, our radiologist and research assistant performed manual segmentation; automatic segmentation was not used in our study. The manual segmentation technique is widely used in current T2 mapping studies. To minimize the effects of manual segmentation, we defined the ROI based on the angle and anatomical landmarks and used the mean values of the two independent measurements for statistical analysis. Further study comparing conventional and FS T2 maps is warranted using automated segmentation method for determination the boundary of articular cartilage. Third, we measured full-thickness cartilage T2 values and did not take into account zonal variation. The effect of chemical shift artifacts depends on the depth of the articular cartilage, which could affect the FS T2 mapping results when the cartilage layers are considered. It is difficult to obtain consistent T2 values from layer by layer due to the spatial resolution of the imaging technique and the thin cartilage thickness. Moreover, evaluation of full-thickness cartilage T2 values may be more practical and reliable in the clinical setting. Fourth, the magic angle effect and partial volume artifacts might have affected the T2 value. Our results showed a bimodal distribution of T2 values in the MFC and LFC with peaks at approximately ± 55°, as in previous T2 mapping studies affected by the magic angle effect [19, 20]. Two-dimensional imaging-based T2 mapping leads to inevitable partial volume averaging artifacts resulting from spatial resolution limitations.

## Conclusions

The T2 values of knee femoral cartilage are significantly lower on FS T2 mapping than on conventional T2 mapping. Additionally, FS T2 mapping is a more reproducible method for evaluating knee femoral cartilage.

## Data Availability

The datasets generated and/or analysed during the current study are not publicly available due to limitations of ethical approval involving the patient data and anonymity but are available from the corresponding author on reasonable request.

## Abbreviations

MR: Magnetic resonance
FS: Fat-suppressed.
MFC: Medial femoral condyle
LFC: Lateral femoral condyle
ROI: Regions of interest
ICC: Intraclass correlation coefficient
